# Prediction of risk of death in severely injured patients: the revised injury severity classification score, version 3 (RISC III)

**DOI:** 10.1007/s00068-026-03224-2

**Published:** 2026-06-08

**Authors:** Rolf Lefering, Sebastian Imach, Dan Bieler

**Affiliations:** 1 Institute for Research in Operative Medicine (IFOM), University of Witten/Herdecke, Ostmerheimer Str. 200 (Building 38), Cologne, 51109 Germany; 2 Department of Trauma and Orthopaedic Surgery, University of Witten/Herdecke, Cologne-Merheim Medical Center, Cologne, Germany; 3 Department of Trauma Surgery and Orthopaedics, Reconstructive Surgery, Hand Surgery and Burn Medicine, German Armed Forces Central Hospital, Koblenz, Germany; 4 Department of Orthopaedics and Trauma Surgery, University Hospital Düsseldorf, Medical Faculty of Heinrich-Heine-University, Düsseldorf, Germany; 5 Committee on Emergency Medicine, Intensive Care and Trauma Management (Sektion NIS, German Trauma Society (DGU), Cologne, Germany

**Keywords:** Trauma registry, Severe injuries, Risk of death, Prognosis

## Abstract

**Purpose:**

Trauma Registries with a focus on severely injured patients use survival as their primary outcome. In order to compare hospitals, interventions, and changes over time, a precise risk of death prediction is mandatory. The German TraumaRegister DGU^®^ uses the RISC II model (Revised Injury Severity Classification, version II) since 2013. The aging trauma population and an improved handling of missing data required the present revision.

**Methods:**

A total of 53,738 seriously injured trauma patients documented in 2022–2023 served as basis for development and validation (3:1 ratio). Missing values should now be considered to be within normal physiological range except in patients with specific findings indicative for an altered physiology. These findings were suggested by clinical experts and validated by registry data. The increasing age of trauma patients was addressed by additional categories and higher point weights. A logistic regression analysis provided new point weights for all predictors. Precision (observed versus predicted mortality) and discrimination (area under the receiver operating characteristic curve, AUROC) were calculated in the development and validation dataset.

**Results:**

Patients in both datasets were well comparable, with a mean age of 55 years, 69% males, and an average Injury Severity Score (ISS) of 18 points. The rate of missing values ranged from 0% (compulsory data) to 17.5% (initial base excess). Missing pre-injury health status was imputed by age, missing pupil size, light reaction, and motor function were imputed by severity of head injury. In case of specific findings, blood pressure and initial laboratory values were imputed by injury severity (ISS), or blood transfusion, or catecholamines, or intake of anticoagulation drugs. The AUROC was 0.946 (95% confidence interval 0.944–0.949) for the new RISC III score which was confirmed in the validation data (0.949; CI 0.945–0.954). Observed and predicted mortality were 13.1% / 13.0% in the development dataset, and 13.2% / 13.0% in the validation dataset.

**Conclusion:**

Risk of death estimates require repeated validations. The increasing number of elderly trauma patients, some of them with restrictions regarding the intensity of treatment, required this update of the RISC II model. New point weights for age were established now, especially for the elderly, in order to enhance the precision of prediction in this patient group. Patients with missing values showed on average a low injury severity, thus replacing a missing value with the normal category as a general rule (RISC III) seems to be superior than replacing it with an average value (RISC II). The new prediction model shows high discrimination and precision in both datasets, development and validation, and will replace the previous version in quality reports and scientific analyses.

## Introduction

Risk of death estimation is a key element in both, quality assessment and scientific analyses, in severely injured patients based on registry data. In quality assessment, the observed mortality rate of a group of patients is compared to a prognosis determined as an average from their individual risk of death estimates. If the observed mortality rate is higher, or lower, than the prognosis, the performance is better, or worse, than expected. This strategy could be applied over time, in a region or country, or when outcome is compared across different hospitals. A high mortality rate is not necessarily bad, it may even characterize a good performance if the expected mortality rate is even higher.

In scientific analyses of large databases and registries, risk of death estimation allows to compare patient groups, or interventions, with uneven mortality rates. Unlike matched pairs, or subgroup analyses, or propensity score matching, it is not the aim to create comparable subsets of patients, but to include all cases, by comparing observed and expected mortality rates [1].

However, risk of death estimation is only reasonable if the applied prognostic instrument or score is highly precise and distinguishes well between survivors and non-survivors. Furthermore, such prediction tools need repeated re-evaluations in order to compare actual mortality rates with the calculated prognosis.

The trauma registry of the German Trauma Society (TraumaRegister DGU^®^, TR-DGU) collects data from severely injured patients since 1993. Initially the Trauma and Injury Severity Score (TRISS [2]) was used as prognostic tool before, in 2009, the first own prognostic score has been developed and published: the Revised Injury Severity Classification (RISC) [3]. In 2014, based on a much larger sample (*n* = 30,886), an updated version was developed and validated, the RISC II [4]. This score has been used since then for the annual audit reports provided for > 700 hospitals, and for numerous scientific publications based on registry data (about 30 per year).

**Fig. 1:**
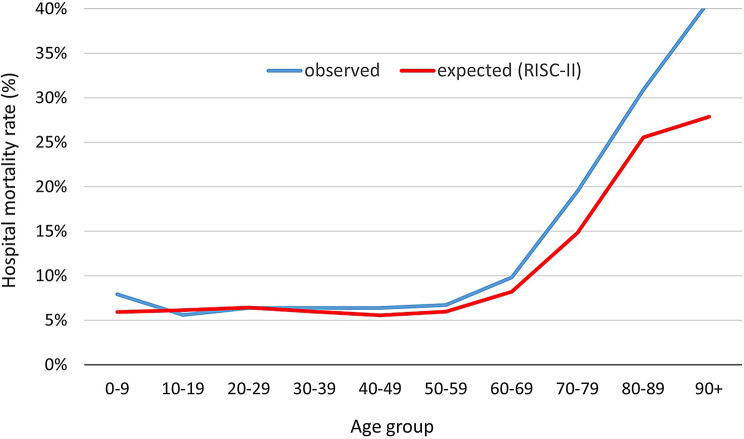
Observed mortality and risk of death prediction based on RISC II, in different age groups, in all 53,738 patients

However, in recent years we observed that risk of death prediction increasingly became imprecise, especially in elderly trauma patients. More elderly patients died than expected (Fig. 1). In an aging society like in Germany, also the average age of trauma patients increased steadily [5], and more than 30% of patients were actually aged 70 years or older [6, 7]. This trend is still ongoing although isolated hip fractures are not included in TR-DGU but contribute to a separate registry (AltersTraumaRegister DGU^®^) [8].

Especially in the elderly, there is also an increasing number of cases who deny or restrict certain interventions. However, the extent of such limitations varies considerably reaching from ‘*no intensive care*’ and ‘*do not resuscitate*’ to ‘*no prolongation of therapy in hopeless situation*s’. Only after exclusion of cases who had such a declaration and died early, the performance of RISC II was still reasonable. However, it was not our aim to exclude more than half of the geriatric non-survivors from quality assessment. Thus, it was our first aim to recalculate the age points to adequately predict the outcome of elderly trauma patients.

The way of how to deal with missing values was the second aim of the present update. In RISC II, a missing value should not change the prediction by receiving zero points in the formula. Positive points increase the chance of survival, while negative points increase the risk of death. Zero points were thus a kind of average effect. This would be adequate if missing occurs at random. However, we had some accidental findings that missing data did not occur at random. For example, Glasgow Coma Scale, pupil size, and light reaction was missing more frequently in patients without head injury. Or base excess was often missing in minor trauma cases without intubation.

Although the general completeness was high – only 0.8 out of 15 components of the RISC II were missing on average [6] – handling of missing values could be improved. Other documented findings which are associated with the missing value could be used to impute the missing one. The knowledge of clinical experts was used to suggest appropriate imputations, and the degree of association could be verified from those cases who had both data available. Such replacement strategies should be implemented for all score components.

Finally, the existing point weights in the prediction formula should be reassessed and adapted, as well as the categories used in the model.

The final version III of the RISC prediction model should then become the standard tool for risk of death prediction in the next years.

## Methods

### TraumaRegister DGU^®^

The TraumaRegister DGU^®^ (TR-DGU) of the German Trauma Society (Deutsche Gesellschaft für Unfallchirurgie, DGU) was founded in 1993. The aim of this multi-center database is a pseudonymized and standardized documentation of severely injured patients.

Data are collected prospectively in four consecutive time phases from the site of the accident until discharge from hospital: (A) Pre-hospital phase, (B) Emergency room and initial surgery, (C) Intensive care unit and (D) Discharge. The documentation includes detailed information on demographics, injury pattern, comorbidities, pre- and in-hospital management, course on the intensive care unit (ICU), relevant laboratory findings including data on transfusion, and outcome of each individual. The inclusion criterion is admission to hospital via the emergency room (trauma team activation) with subsequent intensive or intermediate care. Patients who reached the hospital with vital signs but died before admission to ICU were included as well.

The infrastructure for documentation, data management, and data analysis is provided by AUC - Academy for Trauma Surgery (AUC - Akademie der Unfallchirurgie GmbH), a company affiliated to the German Trauma Society. The scientific leadership is provided by the Committee on Emergency Medicine, Intensive Care and Trauma Management (Sektion NIS) of the German Trauma Society. The participating hospitals submit their data pseudonymised into a central database via a web-based application. Scientific data analysis is approved according to a peer review procedure laid down in the publication guideline of TR-DGU.

The participating hospitals are primarily located in Germany (90%), but a rising number of hospitals of other countries contribute data as well (presently Austria, Belgium, Finland, Luxembourg, Slovenia, Switzerland, The Netherlands, and the United Arab Emirates). Currently, approx. 30,000 cases from over 650 hospitals are entered into the database per year. Participation in TR-DGU is voluntary. For hospitals associated with TraumaNetzwerk DGU^®^, however, the entry of at least a basic data set is mandatory for reasons of quality assurance.

This study was conducted according to the publication guideline of the TR-DGU and registered as project number 2024-025.

### Patients

All patients admitted in 2022 and 2023 qualified for analysis. The minimum injury severity is based on the inclusion criteria of TR-DGU (admitted alive with trauma team activation, and need for intensive care) and the base collective used for the audit reports (maximum AIS ≥ 3, and maximum AIS = 2 only in case of ICU treatment or death, and age available). Patients transferred in from other hospitals were excluded because pre-treatment in another hospital may have changed the physiology on admission. Patients transferred in from another hospital had a better than predicted outcome, as recently has been shown by Halvachizadeh et al. [9]. Patients transferred out early (< 48 h) to another hospital were also excluded since their final outcome was considered unknown.

The total patient group (*n* = 53,738) was split by random into two subgroups for development (*n* = 40,238) and validation (*n* = 13,455), with a 3:1 ratio.

### Data

All injuries were coded according to the Abbreviated Injury Scale (AIS) version 2005; update 2008. The TR-DGU uses a reduced version of the AIS where codes for the same injured structure but with identical severity level were merged. This results in a list of 450 different injuries. User of the online data collection software could use a help function to identify the correct AIS code with a few clicks.

Hospital mortality was used as primary outcome instead of 30 days mortality because no follow-up was performed for those cases discharged alive before day 30.

The independent predictor variables were identical with those of the RISC II model [10], see also Table 1. Specifically, first measurement after admission to hospital should be used. If blood pressure on admission was missing, pre-hospital value is used. If motor reaction (derived from the Glasgow Coma Scale, GCS) and pupils size and reactivity were missing on admission, or patient was sedated or intubated, pre-hospital values are used instead.

## Statistics

Descriptive analysis used number of patients and percentage for categorical data, and mean with standard deviation (SD), or median with quartiles, as appropriate, for metric data. All calculations were performed with SPSS statistical software (version 29; IBM Inc., Armonk, NY, USA).

In contrast to the RISC II model, a category with ‘normal’ physiology was selected for each predictor in the model. This category served as the reference category receiving zero points in the new model. Deviations from normal physiology then will receive negative point weights depending on their importance for risk of death prediction.

Basically, a missing value of a predictor is considered being within the normal physiological range, except that other data of a patient suggest that the (missing) predictor value might not be physiological. For a few predictor variables (age, injury severities), completeness was mandatory, so no replacement was necessary there. For the other predictor variables the rate of missing data is reported, and plausible imputations were suggested by experienced clinicians (DB, SI). This approach was also discussed at the annual meeting of the TR-DGU steering group of Sektion NIS. Categories of the replacement variable were then checked against the predictor variable in patients who had both data available. If patients with a certain replacement condition showed a similar physiology as the predictor variable, then this replacement condition was used to impute the missing predictor value. For example, if a missing base excess (BE) was suggested to be replaced by injury severity (ISS), then for subgroups of patients with high ISS the mean BE was calculated, and if the mean BE fits well into one of the BE categories in the model, high ISS was used to impute a missing BE. If none of the suggested replacement conditions apply, normal physiological values were assumed for the predictor variable in case of missing data. The category of normal values will receive zero points in the final model (reference category). The mortality rate of cases in whom normal values were imputed was cross-checked against the mortality rate of cases with measured normal values. This imputation strategy does not aim to estimate an exact value in case of missing data, but to select an appropriate category.

Finally, a logistic regression analysis was calculated with hospital survival as dependent variable. The predictor variables are the same fifteen predictors used in RISC II, but now with respectively imputed categories for all missing values, as described above. The reference category was always chosen to be the category with physiological values, or in case of binary data the larger category. Based on the coefficients of the final model, score points with one decimal were derived by rounding. Nagelkerke’s R² (range 0–1.0) was provided as pseudo-r-squared metric for the explained variance of the model.

The RISC III score is calculated by summing up the score points from all 15 predictors (including the constant value). Using the logistic function, the sum of score points is transformed into a probability of survival. Risk of death is just 1 minus probability of survival, if required. This procedure is identical to the previous RISC II score.

The area under the receiver operating characteristic (ROC) curve with 95% confidence interval was calculated as a measure of discrimination. The overall precision is demonstrated by comparing observed and predicted mortality in all patients. Calibration (goodness of fit) describes the precision in subgroups of low, medium and high risk patients; it was analysed by calculating observed mortality rates in ten risk bands. Calculations were performed for both scores, RISC II and RISC III. The same calculations were repeated in the validation dataset.

## Results

A total of 53,738 severely injured patients admitted to one of 724 participating hospitals in 2022 and 2023 (83% from Germany) qualified for analysis. 75% of patients were used to develop the new RISC III, and the remaining cases were used for validation. Development and validation datasets were well comparable (Table 2).

**Table 1:** Patient characteristic in the development and validation dataset

	Development 40,283 (75%)	Validation 13,455 (25%)
Age (years)	54.7 (SD 22.6)	54.3 (SD 22.6)
Males	27,899 (69.3%)	9,338 (69.4%)
Traffic accidents	19,032 (47.2%)	6,318 (47.0%)
Injury Severity Score, ISS	18.3 (SD 11.5)	18.2 (SD 11.4)
Penetrating trauma	1,679 (4.4%)	543 (4.2%)
Serious head injury (AIS 3+)	14,363 (35.7%)	4,787 (35.6%)
Isolated head injury	5,379 (13.4%)	1,806 (13.4%)
ICU treatment	34,604 (85.9%)	11,547 (85.8%)
Hospital mortality	5,269 (13.1%)	1,746 (13.0%)
Days in hospital #	9 (4–17)	9 (4–17)
Treated in a German hospital	83.1%	83.0%

SD = standard deviation; # median with quartiles; ICU intensive care unit

**Table 2:** Prevalence of missing data for all RISC predictors, based on the development dataset

Predictor	Missing rate	In case of missing data use …
Age	0%	--- (obligatory data)
AIS injury severity (worst, 2nd worst, head)	0%	--- (obligatory data)
Gender	< 0.1%	cat. male
Cardiac arrest / CPR	6.6%	cat. no
Mechanism	5.8%	cat. blunt
ASA	5.8%	cat. 3 if age ≥ 75, cat. normal (1–2) otherwise
Pupil light reaction	11.2%	cat. sluggish if AIS head = 5, cat. fixed if AIS head = 6, cat. normal otherwise
Pupil size	3.5%	cat. anisocoric if AIS head = 5, cat. bilateral dilated if AIS head = 6, cat. normal otherwise
Motor function	6.0%	cat. directed if AIS head = 3–4, cat. non-directed if AIS head = 5, cat. none if AIS head = 6, cat. normal otherwise
Systolic blood pressure, BP	3.5%	cat. 90–110 if blood transfusion, or ISS ≥ 50, or catecholamines cat. normal (≥ 111) otherwise
Coagulation, INR	7.4%	cat. 1.15–1.59 if ISS ≥ 50, or blood transfusion, cat. >1.60 if Vitamin K antagonist, cat. normal (≤ 1.14) otherwise
Hemoglobin, Hb	3.2%	cat. 7.0–11.9 if blood transfusion cat. normal (≥ 12) otherwise
Base excess, BE	17.5%	cat. -4.0 – -8.9 if ISS ≥ 50, or ≥ 4 units of pRBC, or hemoglobin < 7.0 cat. normal (> -4.0) otherwise

cat. = category; INR = international normalized ratio; pRBC = packed red blood cells

### Imputation

The prevalence rates of missing data for each predictor in the development dataset are presented in Table 2. Overall, 56.4% of patients had complete data for all predictor variables, and the average number of missing data was 0.8 per case. Injury severity (worst AIS, second worst AIS, head injury AIS) and age are obligatory variables and thus had no missing data. For a further three variables, the majority rule was used for imputation, as already practiced in RISC II: for missing gender use ‘male’ (69.3%), for missing cardio-pulmonary resuscitation (CPR) after cardiac arrest use ‘no’ (96.3%), and for missing trauma mechanism use ‘blunt’ (95.6%).

*Pre-injury health status* / American Society of Anaesthesiologists (ASA) classification: three categories are used for the RISC: 1/2, 3, and 4, where ASA 1/2 is considered the reference category. It is well-known that the prevalence of pre-existing diseases increases with age. The ASA 1/2 group showed an average age of 48.8 years, while cases with ASA 3 or 4 were 73.6 and 73.1 years old, respectively. In patients < 60 years of age, prevalence of ASA 3/4 is < 15%. Up to an age of 74 years the prevalence is below 40%. In patients aged 75 years or older, the majority had ASA 3 or 4. Thus age ≥ 75 years was used to replace missing ASA with category 3 (ASA category 4 was rather seldom: 1.1%).

*Pupils*: Pupil size and light reaction are included in the RISC model, both with three categories where normal size and brisk reaction are considered as reference categories (normal values). Altered pupils are associated with severe head injury, and therefore the AIS severity of head injury was used for imputation. Without head injury, normal values were assumed. Brisk light reaction was found in 96 − 77% of cases with head injury grade 1–4, with decreasing prevalence. Normal pupil size was also observed in 97 − 82% of cases with AIS head 1–4. Therefore, normal pupils were assumed if head injury severity was AIS ≤ 4. In head injury grade 5, a relevant portion of patients showed altered pupils, and in head injury grade 6 most cases showed bilaterally dilated pupils (38%) and no light reaction (55%).

**Fig. 2:**
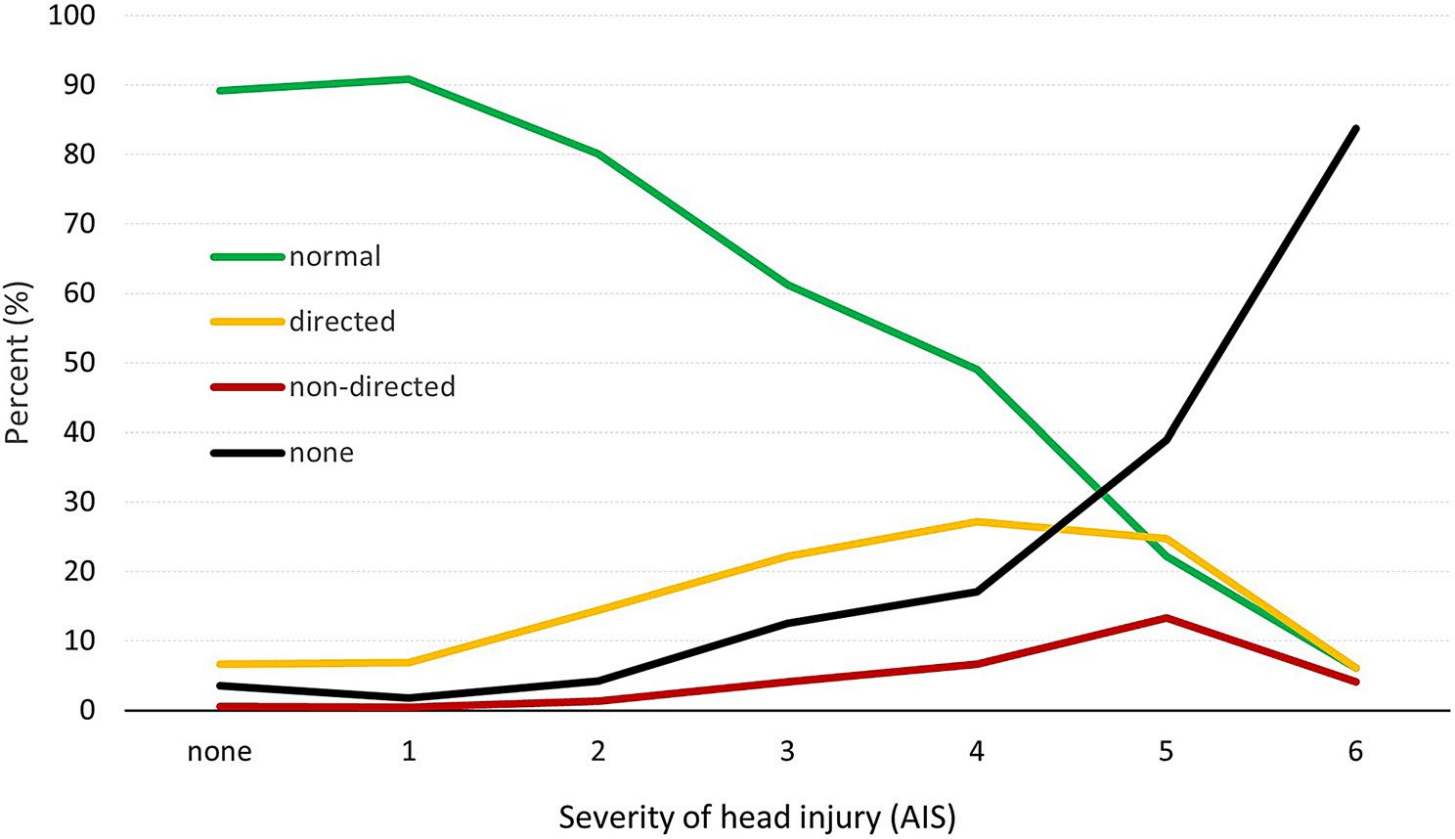
Motor response in patients with a varying severity of head injury (development dataset, patients with available data on motor response)

*Motor response*: Motor response is used with four categories in the RISC model where normal reaction is the reference category. This predictor is part of the Eppendorf-Cologne Scale (ECS) and is a simplification of the 6-level motor component of the Glasgow Coma Scale (GCS) [10]. As for pupils, the association with severe head injury is obvious. In cases without or with only mild (AIS 1–2) head injury a normal motor response was observed in > 80% of cases (Fig. 2). With AIS head 3 an increasing portion of patients showed a non-normal reaction. With AIS head 5/6, the absence of any reaction was the most frequent observation. This led to the decision to replace a missing motor response by ‘normal’ if AIS head was 0–2, ‘directed’ if AIS head was 3–4, ‘non-directed’ if AIS head was 5, and ‘none’ if AIS head was 6.

*Base excess*: Base excess (BE, normal values around − 2 mmol/L), or base deficit (BD, normal values around 2 mmol/L), showed the highest rate of missing data (17.5%). Rather high (BE > 15, 0.1% of values) or low (BE < -30, 0.1%) values were potentially wrong and were thus also considered missing. A BE greater than − 6 (up to + 5) was considered as normal range and served as reference category. Patients with a missing BE had a low mean ISS (15.9 points), and 89% of them were not intubated on admission. This indicated a normal BE in cases with missing data. However, the following situations were associated with a relevant decrease in BE: a high injury severity (ISS ≥ = 50) with an average BE of -8.6 mmol/L, blood transfusion of 4 or more units of packed red blood cells (pRBC) with an average BE of -8.0 mmol/L, and a rather low haemoglobin level (< 7.0 g/dL) with an average BD of -10.0. If one of these conditions were given, the BE category -6.0 to -8.9 was assumed. In all other cases a missing BD was replaced by the reference category.

*Haemoglobin*: Haemoglobin (Hb) was missing in only 3.2% of cases. Values ≥ 12.0 g/dL were considered as normal values and served as reference category. Massive prehospital volume administration (1500 ml or more) was not appropriate for imputation; the mean Hb was 11.9 g/dL. However, patients who required blood transfusion in the emergency room showed an average Hb of 10.6 mg/dL. Thus, the Hb category 7.0–11.9 served as imputation in cases with blood transfusion, while all other cases with missing Hb received the reference category.

*Coagulation / INR*: The International Normalized Ratio (INR) was used to describe coagulopathy on admission, with values < 1.20 considered as normal in RISC II (reference category). Missing data were observed in 7.2% of cases. Besides specific drugs, relevant blood loss was the primary reason for an impaired coagulation. Thus, pre-hospital volume (1500 ml or more; mean INR 1.26), need for early blood transfusion (mean INR 1.38), or need for 4 + units of pRBC (mean INR 1.50), or high injury severity (ISS ≥ 50; mean INR 1.46) were suggested for imputation. Finally, only ISS ≥ 50 and blood transfusion were used to impute the INR category 1.40–2.40. A small group of elderly patients with regular intake of vitamin K antagonists (1.5%) showed a rather high mean INR with 2.49. These cases were imputed by the > 2.40 category of INR. Regular intake of other drugs (anti-platelet drugs, direct oral anticoagulants, heparin) did not lead to an average INR > 1.40.

*Blood pressure*: Already in version II of the RISC score, missing systolic blood pressure (BP) on hospital admission (17.6% of cases) was replaced by first blood pressure measured on scene. The missing data rate could thus be reduced to 3.5%. The range of 111–150 mm/Hg was considered as reference category of normal values. A low BP is often associated with a relevant blood loss. Patients who needed an early blood transfusion (emergency room) showed a mean BP of 106 mmHg. Prehospital need for catecholamine therapy was associated with a mean BP of 107 mmHg. Low blood pressure was also associated with rather high injury severity: if ISS was 50 or higher, mean BP was < 100 mmHg. In the subgroup ISS 35–49 mean blood pressure was 116 mmHg. Thus missing BP was imputed by normal values (category 111–150 mmHg) except for the following conditions: pre-hospital catecholamine therapy, need for early blood transfusion, and ISS ≥ = 50 points. These cases were imputed with the 90–110 mmHg category of BP.

Table 3 summarizes the replacement rules in case of missing data.

**Table 3:** Final model for RISC III, with hospital mortality as dependent variable. The RISC III score has been derived from the regression coefficient (Ref = reference category)

Predictor	Category	Prevalence (%)	Odds Ratio with 95% CI	Coeff.	RISC III score
Worst injury (AIS)	Ref: 2–3 4 5 6	62.3 21.3 15.9 0.6	-- 1.98 (1.76–2.23) 3.23 (2.73–3.83) 16.9 (9.12–31.2)	-- 0.683 1.174 2.825	0 − 0.7 − 1.2 − 2.8
Second worst injury (AIS)	Ref: 0–2 3 4 5	55.1 32.5 9.4 3.1	1.47 (1.32–1.63) 2.18 (1.89–2.50) 3.32 (2.70–4.09)	0.384 0.777 1.200	0 − 0.4 − 0.8 − 1.2
Head injury (AIS)	Ref: 0–2 3–4 5–6	64.3 25.2 10.5	1.10 (0.98–1.24) 2.28 (1.91–2.72)	0.096 0.825	0 − 0.1 − 0.8
Age (years)	Ref: 16–54 0–15 55–59 60–64 65–69 70–74 75–79 80–84 85–89 90+	42.4 3.3 8.9 8.8 6.9 6.5 6.2 8.4 5.7 3.0	0.68 (0.46-1.00) 1.60 (1.29–1.98) 2.59 (2.13–3.14) 3.32 (2.72–4.05) 5.96 (4.95–7.17) 9.92 (8.30–11.9) 14.8 (12.6–17.5) 21.0 (17.6–25.0) 37.8 (30.8–46.4)	-0.391 0.469 0.951 1.201 1.785 2.295 2.697 3.043 3.633	0 + 0.4 − 0.5 − 0.9 − 1.2 − 1.8 − 2.3 − 2.7 − 3.0 − 3.3
Sex	Ref: males Females	69.3 30.7	0.80 (0.73–0.88)	-0.218	0 + 0.2
ASA	Ref: 1–2 3 4	77.0 21.9 1.1	1.75 (1.58–1.93) 3.88 (3.01–5.02)	0.555 1.357	0 − 0.5 − 1.4
Penetrating	Ref: no Yes	96.0 4.0	1.45 (1.15–1.82)	0.370	0 − 0.4
Pupil size	Ref: normal anisocoric both dilated	89.4 6.4 4.2	1.25 (1.09–1.45) 2.37 (1.95–2.89)	0.227 0.864	0 − 0.2 − 0.8
Light reaction	Ref: normal sluggish fixed	87.4 8.1 4.5	1.60 (1.40–1.82) 4.80 (3.94–5.83)	0.467 1.568	0 − 0.5 − 1.6
Motor response	Ref: normal directed non-directed none	73.4 14.2 3.4 9.0	2.38 (2.13–2.67) 3.03 (2.53–3.63) 4.50 (3.88–5.22)	0.868 1.109 1.504	0 − 0.9 − 1.1 − 1.5
CPR	Ref: no Yes	96.9 3.1	5.71 (4.60–7.08)	1.741	0 − 1.7
Systolic BP (mmHg)	Ref: 111+ 90–110 < 90	81.2 13.1 5.7	1.27 (1.13–1.43) 2.37 (2.04–2.75)	0.240 0.862	0 − 0.2 − 0.8
Coagulation, INR	Ref: ≤ 1.14 1.15–1.59 ≥ 1.60	77.1 18.4 4.5	1.42 (1.28–1.56) 1.82 (1.56–2.11)	0,347 0.596	0 − 0.3 − 0.6
Hemoglobin, Hb	Ref: ≥ 12.0 7.0-11.9 < 7.0	75.5 23.2 1.3	1.34 (1.22–1.48) 1.80 (1.36–2.38)	0.295 0.587	0 − 0.3 − 0.6
Base excess, BE	Ref: -> -4 -4 to -8.9^#^ -9 to -14.9 ≤ -15	79.9 14.9 3.2 1.9	1.37 (1.23–1.53) 1.82 (1.51–2.20) 5.13 (4.02–6.55)	0.317 0.600 1.635	0 − 0.3 − 0.6 − 1.6
Constant				-6.02	6.0

Ref = reference category; AIS = severity acc. to the Abbreviated Injury Scale; ASA = American Society of Anaesthesiologists pre-injury health status; CPR = cardio-pulmonary resuscitation; BP = blood pressure; # including BE > 5.0

### Model update

After having imputed all missing data according to the above described procedure, a logistic regression analysis with hospital mortality as dependent variable was computed. For all variables, the category of normal values was used as reference group (for binary predictors, the larger group was used as reference). The first model used the same categories as of RISC version II. The final RISC III is presented in Table 3. Nagelkerke’s R² was 0.625. Remodelling of categories led to the following changes when compared to version II of RISC:

- Worst injury: the reference group now is AIS 2–3 since AIS 2 is too small to serve as a valid reference category (0.6%).
- Age: all children up to the age of 15 years showed similar beneficial effect and formed a new category 0–15 years. The age category 85 + was split into 85–89 and 90+, with an even worse effect for the 90 + category.
- Blood pressure: The previous category > 150 mmHg showed a rather small effect and was merged with the reference category.
- INR: the two worst categories were merged into one new category (INR ≥ 1.60); the threshold of ≥ 1.20 was lowered to ≥ 1.15; reduction from 4 to 3 categories.
- Base excess: moderate values from − 4 to -6 will now be included in the category − 4 to -9 (previously − 6 to -9) due to a similar effect on outcome; rather high values (> 5) will also be included in this new category.

The RISC III score for each category had been derived by appropriate rounding of the regression coefficient. Each category of normal values received zero points. For an individual patient, the sum X of all RISC III scores per predictor, including the constant, is thus considered as a value of the logistic function and could be transformed into a probability of survival by the following formula:

$$P\,\left({survival} \right)\, = \,exp\left(X \right)\,/\,{\rm{ }}\left({1\, + \,exp\left(X \right){\rm{ }}} \right)$$

Where exp(x) is the exponential function ***e***^X^ with Euler’s constant ***e*** = 2,71828…. Risk of death is thus 1 – P(survival) and could be presented as percentage if multiplied by 100.

### Quality of prediction

Discrimination and precision of both, the RISC II and the newly developed RISC III, were calculated in the development and the validation dataset.

The area under the ROC curve, as a cumulated measure for discrimination, has only slightly improved for the new RISC III (AUC 0.946 versus 0.944). However, the precision showed a marked improvement. The version II of the RISC showed a deviation of about 2% in both datasets while the RISC III predicted the observed mortality with only ± 0.2% deviation (Table 4).

**Table 4:** Quality of prediction of RISC II and RISC III, calculated separately in the development and the validation dataset

	Development dataset *n* = 40,283	Validation dataset *n* = 13,455
**Discrimination**	Area under the ROC curve,with 95% confidence interval	
RISC-II	0.944 (0.941 − 0.947)	0.947 (0.942 − 0.952)
**RISC-III**	**0.946 (0.944 – 0.949)**	**0.949 (0.945 − 0.954)**
**Precision**	Observed versus predicted mortality	
Observed hospital mortality	13.1%	13.0%
Expected mortality based on RISC-II	11.0%	11.2%
**Expected mortality based on RISC-III**	**13.0%**	**13.2%**

Patients with complete data for all predictors showed a similar discrimination (AUC 0.946, 95% CI 0.941–0.950) as patients with at least one replacement of missing data (AUC 0.947, 95% CI 0.943–0.951). However, if more than 5 predictors were missing (1.1% of all patients), the RISC III prognosis underestimates the observed mortality by more than 5%.

**Fig. 3:**
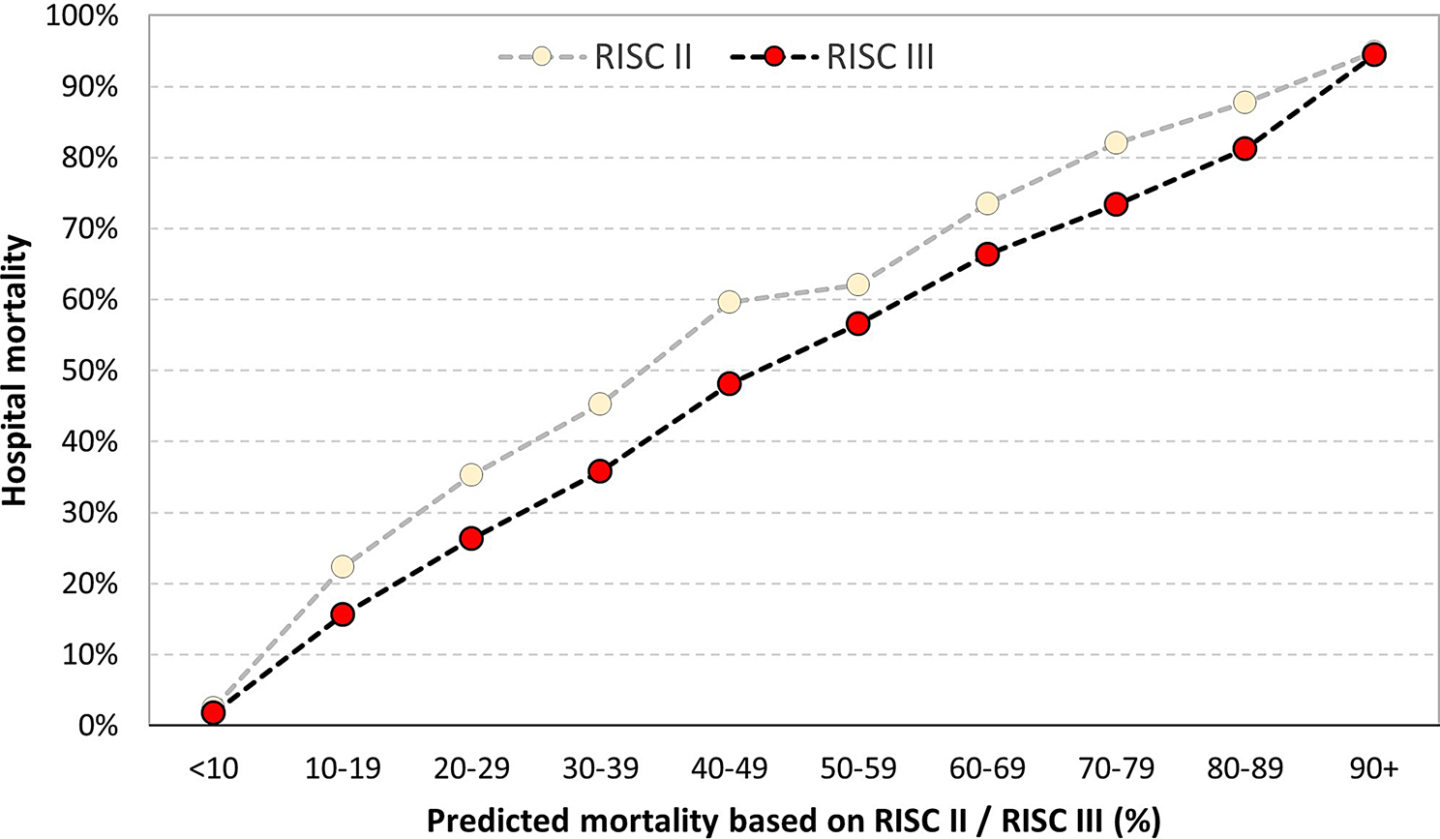
Calibration: observed mortality in ten subgroups of predicted risk of death based on RISC II and RISC III, respectively (development dataset)

Figure 3 shows the observed mortality in ten subgroups of predicted mortality. The first risk band (0-9.9% risk of death) contain 74.8% of all cases. The nearly linear increase demonstrates a good calibration over the whole range of severity estimates. For the RISC II model, the calibration was suboptimal, with higher observed mortality in several subgroups (Fig. 3).

## Discussion

A rare event has led to an intensive discussion about limited therapy in elderly patients: a participating hospital reported that, within a short period of time, there were two admissions of elderly patients, both with serious head injuries after low falls, and in both cases the relatives of the victims denied intensive care. Consequently, both cases died within a few hours. It was agreed that those cases should not be used to quantify the hospital’s quality of care, which is measured as observed versus predicted mortality in TR-DGU. As a consequence, it was decided to add the item ‘therapy-limiting patient will’ in the registry, since 2015. In the subsequent years, patients with a ‘therapy-limiting patient will’, who died within a few days after admission (1 week), were excluded from calculation of a standardized mortality ratio (SMR; observed divided by expected mortality rate).

Since such declarations of patients were mostly found in the elderly, an increasing number of non-survivors were excluded from the evaluation of mortality. In cases aged 75 or above, such declarations were present in more than half of all non-survivors. But we felt that excluding the elderly from quality assessment is no option. Termination of treatment is multifactorial decision, including the clearly stated patient will, health status and development, expected outcomes including quality of live, ethical considerations, and futility of interventions. But it is beyond the scope of trauma registration to adequately measure (and adjust for) all these aspects. Therefore, after 10 years of data collection and experience with the documentation of the ‘therapy-limiting patient will’, the steering group of the TR-DGU decided to change the process, based on the following reasons: (1) the ‘therapy-limiting patient will’ is rather heterogeneous and includes serious limitations like ‘no intensive care’ as well as weak limitations like ‘no prolongation of therapy in futile situations’; (2) patient with such a declaration had an even longer mean hospital stay than those without; (3) the number of excluded patients continuously increased over time; and (4) quality assessment of trauma care should include all treated cases.

This decision, however, required an adaptation of the RISC II score. While the performance of the RISC II was still reasonable after the above mentioned exclusions, it seriously under-estimated the risk of death especially in the elderly if all cases were included (Fig. 1). It was decided not to include the ‘patient will’ as an additional predictor, due to its heterogeneity, but to incorporate the increased risk of death in elderly by increasing the age points in the score.

The second reason for the actual revision of the RISC II model was the management of missing data. In a recent review about publications from trauma registries, Shivasabesan et al. found that mainly complete case analyses were performed, excluding cases with missing data, or authors did not even address the problem of missing data [11]. Missing data is a permanent problem in registries since no source data verification is possible, like in prospective clinical trials [12]. Fortunately, the portion of patients with missing data in the fifteen core variables needed for risk of death prediction is relatively low (see Table 2). However, missing data may bias the outcome prediction. The previous approach in RISC II did not add points to the score if an information was missing (0 points). The rationale behind this was that a missing information should not influence the prediction. Since categories in RISC II received either positive scores (in case of normal physiological values, e.g. blood pressure 111–150 mmHg) and negative values (in case of serious deviations, e.g. blood pressure < 90 mmHg), zero points reflect an average impact on mortality (e.g. blood pressure 90–110 mmHg). This approach assumes a missing at random.

However, the inspection of cases with missing data, for each variable, revealed that the majority of cased had a rather low injury severity and mortality. Therefore, the new approach in RISC III will in general consider a missing value to be in a normal physiological range, and only in selected cases, negative points will be given. Normal values will in general receive zero points. This principle had already been used in the first version of the RISC [3]. As part of the formula, the constant value is now 6.0 which corresponds to a 99.8% chance of survival, if no other deviations were found. Nearly all deviations from normal values receive negative points, except for female sex (+ 0.2 points) and young age (+ 0.4 points). The replacement rules for missing data, specifically where normal values were not realistic, were based on suggestions by experienced clinicians with subsequent verification using registry data. It is important to note that this approach was chosen over a formal statistical approach of (multivariable) imputation of missing data since the transparency of imputation is a key element for the acceptance of the RISC models. This continues the general strategy of the RISC models where simple categories were used instead of complex non-linear functions (e.g. for age), and simple coefficients instead of highly precise point weights. A recent evaluation from the Dutch trauma registry published by de Munter et al. could show that ‘simplified imputation models’ (as ours) could adequately be used to impute missing data [13].

Finally, a few adjustments have been made for age, INR, and base deficit. But in general, the same predictors were used in RISC III as in the previous RISC II. Since no new predictor was included in the model, the discrimination (area under the ROC curve) only marginally increased, as expected. However, the precision is much better now: observed and predicted mortality differ by less than 0.2%, both in the development and validation sample. The increased risk of death in the elderly caused by various degrees of limited therapies and increased vulnerability is now incorporated into the model by higher age points.

Coming back to the initial situations where the receiving hospital was not allowed to do any relevant intervention: such patients would now receive a worse prognosis. However, if no adequate initial therapy was possible, those cases should not be considered for quality assessment at all.

### Limitations

Although management of missing data has now been improved, it is not recommended to apply the RISC III prognostic system in datasets where one or more variables were missing completely. In such situations it is recommended to derive a new formula without the missing variable. It is also not recommended to use the RISC III model if more than 5 predictors were missing.

We only applied an internal validation, using a random split of the available population. Given the large number of cases, this approach is expected to yield satisfactory results. Similar results could be expected using future TR-DGU data, at least in the next years. However, as emphasized in the TRIPOD statement [14], an independent validation using data from other registries could further strengthen the validity of RISC III model. As has been performed for the RISC II [15], external cooperations with other trauma registries have been initiated to validate this score. This is facilitated by the fact that RISC III is based on the European Utstein Core Dataset [16].

Furthermore, it has to be acknowledged that 83% of the present patient cohort was treated in Germany, with a traditionally physician-based emergency system. Pre-hospital interventions, like intubation and volume therapy, may have an influence on the patients’ condition on admission. External validations are thus even more important.

## Conclusion

Risk of death estimates used in trauma registries should be validated regularly. The RISC II model showed considerable deviation from observed mortality especially in the elderly. Furthermore, a reasonable imputation of missing values, despite a rather high overall availability of data, might further improve the precision and discrimination of a prognostic score.

The RISC prediction model version III has preserved it’s still high discrimination of survivors and non-survivors, but with a relevant improvement in precision. This score will be used by TR-DGU during the next years for quality assessments and scientific analyses of severely injured patients.

## Data Availability

Raw data from TraumaRegister DGU are not publicly available.
